# Effects of Different Monomeric Bile Acids in the Diet on the Growth and Lipid Metabolism of Juvenile Pacific White Shrimp

**DOI:** 10.3390/ani16121925

**Published:** 2026-06-22

**Authors:** Ziling Song, Yang Liu, Huan Liu, Zhengwei Ye, Lindong Xiao, Qiang Ma, Yuliang Wei, Mengqing Liang, Houguo Xu

**Affiliations:** 1 College of Fisheries and Life Sciences, Shanghai Ocean University, 999 Huchenghuan Road, Shanghai 201306, China; 2 State Key Laboratory of Mariculture Biobreeding and Sustainable Goods, Yellow Sea Fisheries Research Institute, Chinese Academy of Fishery Sciences, 106 Nanjing Road, Qingdao 266071, China; 3 Weifang Key Laboratory of Precise Animal Nutrition, Weifang Kenon Biotechnology Co., Ltd., Weifang 261108, China

**Keywords:** bile acid transport, cholesterol metabolism, sterol metabolism, lipid catabolism

## Abstract

Bile acids are important for lipid digestion and metabolic regulation, but the roles of individual bile acids in crustaceans remain unclear. This study evaluated the effects of six dietary monomeric bile acids on growth and lipid metabolism in juvenile Pacific white shrimp. Juvenile shrimp (2.5 g) were fed a basal diet or the same diet supplemented with 0.04% bile acid (cholic acid, hyodeoxycholic acid, chenodeoxycholic acid, deoxycholic acid, ursodeoxycholic acid, or hyocholic acid) for 8 weeks. Each treatment was assigned to three replicate 100-L tanks, with 30 shrimp per tank. Dietary monomeric bile acids did not significantly affect growth performance, body composition, or muscle fatty acid composition. Compared with the control group, chenodeoxycholic acid, deoxycholic acid, and hyocholic acid significantly reduced the hemolymph triglyceride levels, while cholic acid, chenodeoxycholic acid, and hyocholic acid lowered the hepatopancreatic lipid levels. All bile acid treatments reduced the hepatopancreatic malondialdehyde content compared with the control group. Expression of genes related to bile acid transport, sterol metabolism, and lipid catabolism was generally upregulated by bile acids, indicating enhanced bile acid circulation and lipid turnover. In conclusion, monomeric bile acids mainly regulate lipid metabolism and oxidative status rather than directly promoting growth under the present dietary condition, with chenodeoxycholic acid, cholic acid, and deoxycholic acid showing relatively stronger effects.

## Simple Summary

Bile acids help animals digest lipids and regulate lipid metabolism. However, most studies in aquaculture have focused on mixed bile acids, and the roles of individual bile acids are still unclear, especially in shrimp. In this study, juvenile Pacific white shrimp (*Litopenaeus vannamei*) were fed diets containing six different monomeric bile acids for eight weeks. These monomeric bile acids did not affect the growth, body composition, or muscle fatty acid composition. However, several bile acids lowered the triglyceride and cholesterol levels in the hemolymph and hepatopancreas, reduced the oxidative damage, and increased the transcription of genes involved in bile acid transport, cholesterol turnover, and lipid breakdown. Among the tested compounds, chenodeoxycholic acid, deoxycholic acid, and cholic acid showed stronger effects on lipid regulation than others. In conclusion, different monomeric bile acids did not act in the same way and need be used more precisely in shrimp feeds to support efficient lipid metabolism.

## 1. Introduction

Pacific white shrimp (*Litopenaeus vannamei*) has become a key crustacean species in global aquaculture [1]. In recent years, with the rapid development of intensive aquaculture modes, the demand for efficient and sustainable feed nutritional strategies has continuously increased. Lipid is one of the crucial energy sources for crustaceans and also an important component of cellular structure. Dietary lipids not only provide energy for the organism but also participate in various physiological processes. However, with the expansion of aquaculture scale, traditional fish oil resources are becoming increasingly scarce, leading to the gradual replacement of fish oil with vegetable and animal oils as alternative lipid sources [2,3,4]. Nevertheless, these alternative lipid sources often face issues such as imbalanced fatty acid composition and low digestibility, which can negatively affect the growth and health of shrimp [5,6]. Consequently, improving the utilization efficiency of dietary lipids has become a significant focus in current aquatic nutrition research.

Bile acids (BAs) are a class of steroid compounds produced from cholesterol metabolism and are essential components of bile. BAs not only promote lipid emulsification and enhance fat digestibility, but also act as signaling molecules involved in regulating physiological processes such as lipid metabolism, cholesterol homeostasis, and antioxidant responses. Based on their composition, bile acids are generally categorized into complex bile acids and monomeric bile acids. In the past decade, complex bile acids have been widely used in aqua-feeds and have been demonstrated to improve growth performance, enhance lipid utilization efficiency, and boost antioxidant capacity [7,8,9,10,11,12,13]. However, because mixed bile acid products contain multiple bile acid components, the specific effects of individual monomeric bile acids remain insufficiently understood in aquaculture species, particularly in crustaceans such as Pacific white shrimp.

Over the past ten years, some studies have begun to focus on the role of monomeric bile acids in aquatic animals. Research on monomeric bile acids in crustaceans remains very limited. It is noteworthy that crustaceans have limited ability to synthesize sterols and bile acids de novo and therefore mainly rely on dietary sources to obtain these compounds during growth and development [14,15]. In contrast, studies in fish have shown that different monomeric bile acids may have distinct biological functions. For instance, chenodeoxycholic acid (CDCA) has been shown to significantly promote growth and reduce liver lipid deposition in large yellow croaker (*Larimichthys crocea*) and yellow catfish (*Pelteobagrus fulvidraco*) [16,17]; ursodeoxycholic acid (UDCA) has been reported to improve antioxidant capacity and regulate cholesterol metabolism in grass carp (*Ctenopharyngodon idella*) and juvenile turbot (*Scophthalmus maximus*) [18,19]; and hyodeoxycholic acid (HDCA) can enhance lipid digestibility and utilization while reducing lipid deposition in juvenile *Schizothorax prenanti* [20]. Additionally, cholic acid (CA) and deoxycholic acid (DCA) can improve lipid metabolism by regulating the expression of genes related to lipid synthesis and catabolism in hybrid grouper and grass carp [21,22]. However, whether these effects also occur in crustaceans, especially Pacific white shrimp, remains unclear. Therefore, systematically comparing the metabolic regulatory effects of different monomeric bile acids is of great significance for elucidating their nutritional functions and optimizing feed formulation.

We hypothesized that different monomeric bile acids would have different regulatory effects on lipid metabolism, antioxidant status, and related metabolic gene expression in Pacific white shrimp. Therefore, using Pacific white shrimp as the research model, the present study aimed to systematically compare the effects of six monomeric bile acids (CA, HDCA, CDCA, DCA, UDCA, and hyocholic acid (HCA)). The evaluation included the response of growth performance, body composition, serum biochemical indicators, and the expression of genes related to bile acid metabolism, sterol metabolism, and lipid metabolism. The results may contribute to the precise application of bile acids in shrimp feeds. To our knowledge, this study is the first to systematically compare the metabolic regulatory effects of multiple monomeric bile acids on Pacific white shrimp.

## 2. Materials and Methods

### 2.1. Experimental Diets and Feeding Trial

Seven experimental diets were prepared for the feeding trial (Table 1). The control diet was formulated to contain an appropriate lipid level of approximately 7% for juvenile Pacific white shrimp. Six treatment diets were prepared by supplementing 0.04% different monomeric bile acids (reagent grade, purity > 95%, Shanghai Aladdin Biochemical Technology Co., Ltd., Shanghai, China) into the basal diet. These diets were named according to the supplemented bile acid as follows: cholic acid (CA), hyodeoxycholic acid (HDCA), chenodeoxycholic acid (CDCA), deoxycholic acid (DCA), ursodeoxycholic acid (UDCA), and hyocholic acid (HCA). This supplementation level was selected as a practical baseline dose based on previous studies in aquatic animals using dietary bile acids or monomeric bile acids, and it allowed for the direct comparison of different bile acid types under the same dietary background [16,20,23,24,25]. However, this concentration was not intended to represent the optimal inclusion level for each individual bile acid. During feed preparation, dry ingredients were ground, passed through an 80-mesh sieve, and mixed thoroughly. The oil mixture was then added and blended evenly with the dry ingredients. After water was added, the mixture was pelleted into 1.0-mm pellets using a single-screw pelletizer. The pellets were dried at 55 °C, cooled to room temperature, sealed in bags, and stored at −20 °C until use.

### 2.2. Experimental Shrimp and Feeding Management

The feeding trial was conducted at the Langya Experimental Base of the Yellow Sea Fisheries Research Institute (Qingdao, China). Juvenile Pacific white shrimp with an initial average body weight of 2.5 g were acclimated for two weeks prior to the experiment. During this period, shrimp were fed a commercial diet supplied by Guangdong Haida Group Co., Ltd. (Guangzhou, China). Before the trial, shrimp were fasted for 24 h and then randomly assigned to 21 polyethylene tanks. Each diet was tested in triplicate, with 30 shrimp stocked in each 100-L tank, corresponding to a stocking density of 0.3 shrimp/L. Shrimp were hand-fed to apparent satiation four times daily at 7:00, 12:00, 17:00, and 22:00 for 8 weeks. The culture system was operated under static-water conditions with continuous aeration. Uneaten feed and feces were removed daily by siphoning, and the tanks were cleaned regularly. Approximately two-thirds of the water in each tank was replaced every day. During the trial, water temperature was maintained at 27–31 °C, salinity at 28–30, dissolved oxygen above 8 mg/L, and pH at 7.6–7.9.

### 2.3. Sample Collection

At the end of the feeding experiment, the shrimp were anesthetized with eugenol (1:10,000 water). All shrimp in each tank were counted and weighed for growth performance calculation. Three shrimp were first randomly selected from each replicate tank for whole-body proximate composition analysis. Subsequently, another eight shrimp were randomly sampled from the remaining shrimp in each tank for tissue collection. Hemolymph was first collected from the pereiopods using a 1-mL syringe and immediately mixed with anticoagulant at a ratio of 1:2 (*v*/*v*). The mixture was kept at 4 °C for 4 h and then centrifuged at 4000× *g* for 10 min at 4 °C to obtain the supernatant. After hemolymph collection, the hepatopancreas, muscle, and intestine were dissected from the same shrimp. Hepatopancreas samples were used for biochemical parameter determination and qRT-PCR analysis, while muscle samples were used for proximate composition and fatty acid analysis. All collected tissues were immediately frozen in liquid nitrogen and stored at −80 °C until further analysis. All procedures were approved by the Animal Care and Use Committee of the Yellow Sea Fisheries Research Institute, Chinese Academy of Fishery Sciences.

### 2.4. Analysis of the Proximate Composition and Fatty Acid Composition

The proximate composition of experimental diets and whole shrimp was determined using standard analytical procedures. Briefly, moisture was measured by drying samples to constant weight at 105 °C, crude protein was analyzed using the Kjeldahl method with a FOSS KJELTEC 2300 system (Hillerod, Denmark), crude lipid was extracted using the chloroform–methanol method [26], and ash was determined after combustion in a muffle furnace at 550 °C.

Muscle fatty acid composition was determined by gas chromatography (GC-2010 Pro, Shimadzu, Japan). Muscle samples were freeze-dried for 48 h using a freeze dryer (FDU-1100, Tokyo Rikakikai Co., Ltd., Tokyo, Japan), and total lipid was extracted using the chloroform–methanol method. For fatty acid methyl ester preparation, 30 μL of lipid extract was transferred into a 10-mL glass tube, mixed with 2 mL of 0.5 mol/L potassium hydroxide–methanol solution, and heated at 75 °C for 30 min. After cooling, 1 mL of boron trifluoride–methanol solution was added, followed by another incubation at 75 °C for 30 min. Then, 1 mL of distilled water and 1 mL of n-hexane were added, and the mixture was vortexed and kept on ice for 1 h. The upper organic phase was collected for gas chromatographic analysis. Separation was performed using a fused silica capillary column (SH-RT-2560, 100 m × 0.25 mm × 0.20 μm, Shimadzu, Japan). The injector and detector temperatures were both set at 250 °C. The oven temperature was increased from 150 °C to 200 °C at 15 °C/min and then from 200 °C to 250 °C at 2 °C/min. Fatty acid composition was expressed as the percentage of each fatty acid relative to total fatty acids.

### 2.5. Biochemical Parameters in Hemolymph and Hepatopancreas

Biochemical parameters in the hemolymph and hepatopancreas were measured using commercial assay kits following the manufacturers’ protocols. In the hemolymph, total cholesterol (TC), triglycerides (TG), high-density lipoprotein cholesterol (HDL-C), low-density lipoprotein cholesterol (LDL-C), malondialdehyde (MDA), total bile acids (TBA), hepatic lipase (HL), and lipoprotein lipase (LL) were analyzed. The kits for hemolymph TC, HL, and LL were obtained from Beijing Solarbio Science & Technology Co., Ltd. (Beijing, China), while the remaining kits were purchased from Nanjing Jiancheng Bioengineering Institute (Nanjing, China).

For hepatopancreas analysis, tissue samples were homogenized with either 0.9% saline or an n-heptane mixture at a ratio of 1:1 according to the corresponding kit instructions. The homogenates were centrifuged, and the supernatants were collected for the determination of TC, TG, TBA, and MDA. Kits for hepatopancreatic TC and TG were purchased from Beijing Solarbio Science & Technology Co., Ltd. (Beijing, China), and kits for TBA and MDA were obtained from Nanjing Jiancheng Bioengineering Institute (Nanjing, China).

### 2.6. Quantitative Real-Time Polymerase Chain Reaction (qRT-PCR)

Total RNA was extracted from the hepatopancreas samples, and qRT-PCR was performed using the reagents from Accurate Biotechnology and TsingKe Biological Technology (Qingdao, China) with a Roche LightCycler 96 system (Basel, Switzerland), following our previously established protocols [27]. The primer sequences and primers used in this study are provided in Table 2. Some primers were adopted from our previous publications or other published studies on *Litopenaeus vannamei* [24,25,26,27,28,29,30], whereas primers without available published sequences were designed based on the corresponding nucleotide sequences deposited in the NCBI database. All primers were checked for specificity before use, and single peaks in the melting curves confirmed specific amplification. The amplification efficiency of each primer pair was also verified and was within the acceptable range for qRT-PCR analysis. The relative mRNA expression was evaluated with the 2^−ΔΔCT^ method [31]. The reference genes *ef-1α* and *β-actin* were selected based on previous studies and our established qRT-PCR methods in Pacific white shrimp. The geometric mean of the Ct values of these two reference genes was used for normalization [32].

### 2.7. Biometrics

Body weight, body length, weight gain, and feed conversion ratio of shrimp were measured at the end of the feeding trial. Calculations are according to the following equations:

Weight gain (WG, g) = final weight − initial weight

Weight gain ratio (WGR, %) = (final weight − initial weight)/IBW × 100;

Specific growth rate (SGR, %/day) = (ln final weight − ln initial weight)/experimental days × 100;

Feed conversion ratio (FCR) = feed intake/weight gain;

Survival (%) = final shrimp number/initial shrimp number × 100.

### 2.8. Statistical Methods

All data were subjected to one-way analysis of variance (ANOVA) in SPSS 26.0 for Windows. Tukey’s multiple range test was used to detect the significant differences between the means. The significance was accepted when *p* < 0.05. The results are presented as means of triplicate tanks ± standard error.

## 3. Results

### 3.1. Growth Performances and Somatic Indices

There were no significant differences in final body weight, WG, SGR, FCR, and SR among all groups (*p* > 0.05) (Table 3).

### 3.2. Body Proximate Composition

There was no significant difference in crude protein, crude fat, moisture, and ash of whole shrimp among groups (*p* > 0.05, Table 4) as well as no significant difference in crude protein, crude lipid, and moisture contents in muscle among groups (*p* > 0.05).

### 3.3. Fatty Acid Profiles of the Muscle

There was no significant difference in fatty acid composition among all groups (*p* > 0.05, Table 5).

### 3.4. Biochemical Parameters in Hemolymph and Hepatopancreas

**Figure 1:**
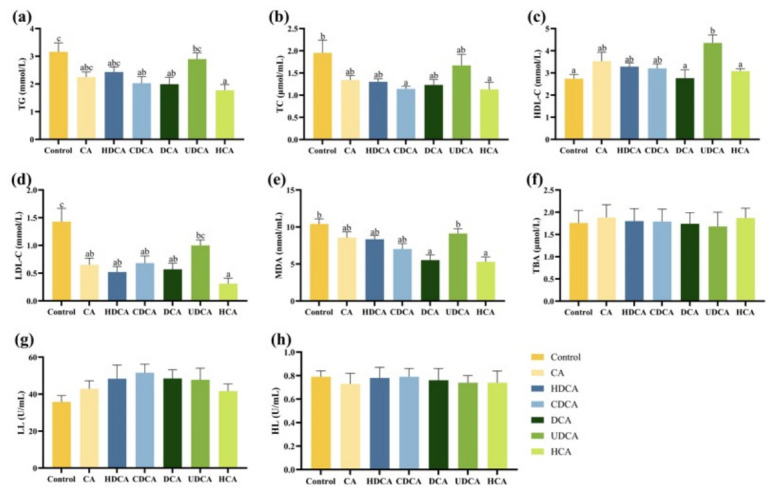
Lipid-related biochemical parameters in the hemolymph of experimental shrimp fed diets containing different bile acids (mean ± standard error). Data bars not sharing a same superscript letter are significantly different (*p* ˂ 0.05). (**a**) TC, total cholesterol; (**b**) TG, triglycerides; (**c**) HDL-C, high-density lipoprotein cholesterol; (**d**) LDL-C, low-density lipoprotein cholesterol; (**e**) MDA, malondialdehyde; (**f**) TBA, total bile acids; (**g**) HL, hepatic lipase; (**h**) LL, lipoprotein lipase.

In the hemolymph, the TG content in the CDCA, DCA, and HCA groups was significantly lower than that in the control group (*p* < 0.05, Figure 1a). Additionally, TG content in the HCA group was lower than in the UDCA group (*p* < 0.05, Figure 1a). The TC content in the CDCA and HCA groups was significantly lower compared to the control group (*p* < 0.05, Figure 1b). The HDL-C content in the control, DCA, and HCA groups was significantly lower compared to the UDCA group (*p* < 0.05, Figure 1c). The LDL-C content in the control group was significantly higher than that in all other groups except the UDCA group (*p* < 0.05, Figure 1d). In addition, the HCA group had significantly lower LDL-C content than the UDCA group (*p* < 0.05, Figure 1d). The MDA content in the DCA and HCA groups was significantly lower compared to the UDCA and control groups (*p* < 0.05, Figure 1e). There were no significant differences in TBA, LL, and HL content among all groups (*p* > 0.05, Figure 1f–h).

**Figure 2:**
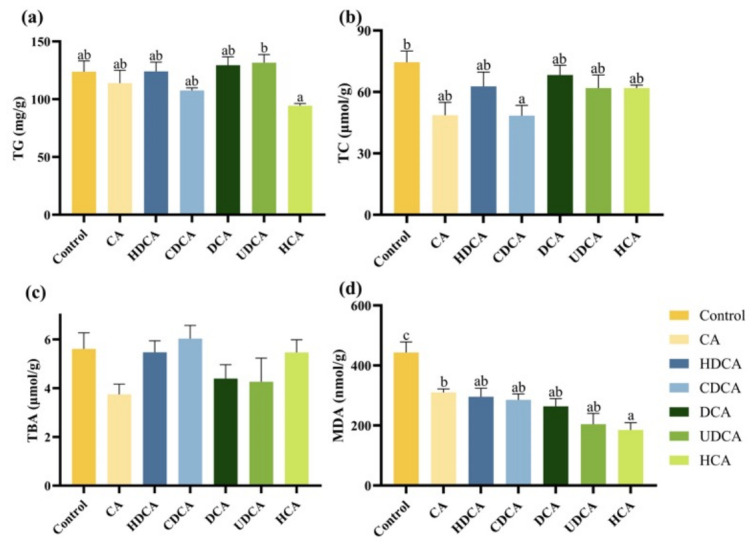
Hepatopancreas of experimental shrimp fed with diets containing different bile acids (mean ± standard error). Data bars not sharing a same superscript letter are significantly different (*p* ˂ 0.05). (**a**) TC, total cholesterol; (**b**) TG, triglycerides; (**c**) TBA, total bile acids; (**d**) MDA, malondialdehyde.

The TG content in the HCA group was significantly lower compared to the UDCA group (*p* < 0.05, Figure 2a). The TC content in the CDCA group was significantly lower compared to the control group (*p* < 0.05, Figure 2b). The MDA content in the control group was significantly higher than that in all other groups (*p* < 0.05, Figure 2d). In addition, the MDA content in the HCA group was significantly lower than in the CA group (*p* < 0.05, Figure 2d). In the hepatopancreas, there were no significant differences in TBA content among all groups (*p* > 0.05, Figure 2c).

### 3.5. The mRNA Expression in Hepatopancreas

#### 3.5.1. Expression of Genes Related to Bile Acid Metabolism

**Figure 3:**
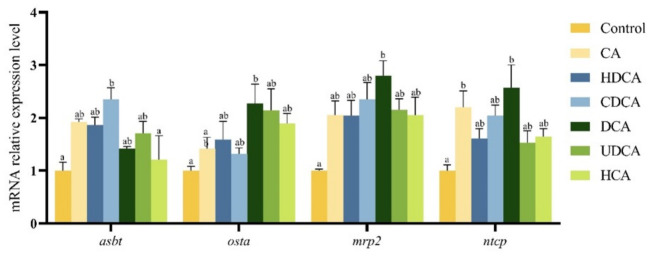
Relative mRNA expression of bile acid metabolism-related genes in the hepatopancreas of shrimp fed diets containing different bile acids (mean ± standard error). Data bars not sharing a same superscript letter are significantly different (*p* ˂ 0.05). *asbt*, sodium/bile acid cotransporter-like; *ostα*, organic solute transporter subunit alpha-like; *mrp3*, multidrug resistance-associated protein 2-like; *ntcp*, sodium taurocholate co-transporting polypeptide.

The expression level of *asbt* in the CDCA group was significantly higher compared to the control and HCA group (*p* < 0.05, Figure 3). The expression levels of *osta*, *mrp3*, and *ntcp* in the DCA group were significantly higher compared to the control group (*p* < 0.05). The expression levels of *asbt*, *osta*, *mrp3*, and *ntcp* in all treatment groups were higher than those in the control group.

#### 3.5.2. Expression of Genes Related to Sterol Metabolism

**Figure 4:**
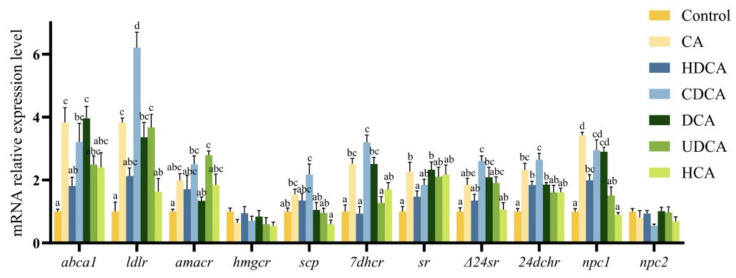
Relative mRNA expression of sterol metabolism-related genes in the hepatopancreas of shrimp fed diets containing different bile acids (mean ± standard error). Data bars not sharing a same superscript letter are significantly different (*p* ˂ 0.05). *abca1*, ATP-binding cassette sub-family A 1; *ldlr*, low-density lipoprotein receptor; *amacr*, 4F2 cell-surface antigen heavy chain-like; *hmgcr*, 3-hydroxy-3-methylglutaryl-coenzyme A reductase-like; *scp*, sterol carrier protein 2; *7dhcr*, 7-dehydrocholesterol reductase-like; *sr*, steroid reductase; *Δ24sr,* delta(24)-sterol reductase-like; *24dchr*, 24-dehydrocholesterol reductase; *npc1*, NPC intracellular cholesterol transporter 1; *npc2*, NPC intracellular cholesterol transporter 2.

The expression levels of *abca1*, *ldlr*, *7dhcr*, *sr*, *24dhcr*, and *npc1* in the CA group were significantly higher than those in the control group (*p* < 0.05, Figure 4). The expression levels of *24dhcr* and *npc1* in the HDCA group were significantly higher than those in the control group (*p* < 0.05). The expression levels of *abca1*, *ldlr*, *amacr*, *scp*, *7dhcr*, *Δ24sr*, *24dhcr,* and *npc1* in the CDCA group were significantly higher than those in the control group (*p* < 0.05). The expression levels of *abca1*, *ldlr*, *7dhcr*, *sr*, *Δ24sr*, *24dhcr*, and *npc1* in the DCA group were significantly higher compared to the control group (*p* < 0.05). The expression levels of *ldlr* and *amacr* in the UDCA group were significantly higher compared to the control group (*p* < 0.05). There were no significant differences in the expression levels of *hmgcr* and *npc2* among all treatment groups (*p* > 0.05).

#### 3.5.3. Lipid Metabolism-Related Gene Expression

**Figure 5:**
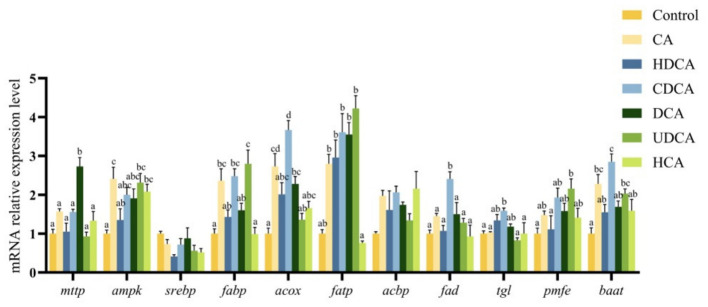
Relative mRNA expression of lipid metabolism-related genes in the hepatopancreas of shrimp fed diets containing different bile acids (mean ± standard error). Data bars not sharing a same superscript letter are significantly different (*p* ˂ 0.05). *mttp*, microsomal triglyceride transfer protein large subunit; *ampk*, AMP-activated protein kinase subunit beta; *srebp*, sterol-regulatory element binding protein; *fabp*, fatty acid binding protein 1-B.1-like; *acox*, peroxisomal acyl-coenzyme a oxidase 1-like; *fatp*, protein unc-13 homolog 4B-like; *acbp*, acyl-CoA-binding protein; *fad*, delta6 fatty acyl desaturase; *tgl*, triacylglycerol lipase; *pmfe*, peroxisomal multifunctional enzyme type 2-like; *baat*, acyl-coenzyme A thioesterase 1-like.

There were no significant differences in the expression levels of *srebp* and *acbp* among all treatment groups (*p* > 0.05, Figure 5), but the expression level of *srebp* in all treatment groups was lower compared to the control group, while the expression level of *acbp* was higher than that in the control group. The expression levels of *ampk*, *fabp*, *acox*, and *baat* in the CA group were significantly higher than those in the control group (*p* < 0.05). The expression levels of *acox*, *fad*, *tgl*, and *baat* in the CDCA group were significantly higher compared to the control group (*p* < 0.05). The expression levels of *mttp* and *acox* in the DCA group were significantly higher compared to the control group (*p* < 0.05). The expression levels of *ampk*, *fabp*, *pmfe*, and *baat* in the UDCA group were significantly higher compared to the control group (*p* < 0.05). The expression level of *ampk* in the HCA group was significantly higher than that in the control group (*p* < 0.05). Except for the control group, the expression level of *fatp* in all groups was significantly higher compared to the HCA group (*p* < 0.05).

## 4. Discussion

The results of this study indicate that under the present experimental conditions, different monomeric bile acids did not have a significant impact on the growth performance of Pacific white shrimp. However, significant effects were found on hemolymph lipid levels, antioxidant capacity, and the expression of genes related to lipid metabolism. CDCA, DCA, and HCA significantly reduced the hemolymph TG content, CDCA and HCA significantly reduced the hemolymph TC content, and CDCA significantly reduced the hepatopancreatic TC content. Moreover, all bile acid treatments reduced the hepatopancreatic MDA content. In studies on other aquatic animals, bile acid supplementation has shown variable effects on growth performance, including growth promotion in some species [33,34,35,36,37,38,39,40] and no significant effects in others [9,28,41].

For crustaceans, studies on monomeric bile acids are very limited, and available evidence has mainly focused on CDCA. In tiger shrimp (*Penaeus monodon*) and Pacific white shrimp, dietary CDCA did not consistently improve growth performance [24,25,42]. Similarly, no growth-promoting effect of CDCA was observed in the present study, suggesting that its effect on growth may be limited in shrimp under the current dietary condition. Although CDCA has been reported to promote growth in large yellow croaker and yellow catfish [16,17], such effects were not observed in some other fish species, such as largemouth bass (*Micropterus salmoides*) and grass carp [22,23].

Previous studies have also reported variable growth responses to other bile acids. UDCA may negatively affect growth in Gibel carp [43] while showing biphasic responses in large yellow croaker [44]. HDCA has been reported to promote growth in Przevalski’s schizothoracin [20]. Overall, these results suggest that the effects of monomeric bile acids on growth performance are strongly species- and context-dependent. In the present study, 0.04% was selected as a commonly used concentration based on previous studies without dose screening, which may also contribute to the observed variability.

This dose allowed for a direct comparison of the six monomeric bile acids under the same dietary background. However, because no dose–response design was included, 0.04% should not be regarded as the optimal inclusion level for each bile acid. Therefore, differences among treatments should be interpreted as relative response patterns under the present single-dose condition rather than a definitive ranking of intrinsic efficacy. Further studies are required to determine the optimal inclusion level and safety margin of each monomeric bile acid in Pacific white shrimp. The lack of a significant growth-promoting effect may be related to the nutritional background of the basal diet (20% fish meal, 1% soya lecithin, and ~7% lipid), which may have already supported normal growth and lipid digestion, thereby partially masking growth-related responses. In addition, species differences in bile acid physiology may explain why shrimp responses are more reflected in lipid transport, sterol turnover, and oxidative status than in growth performance. Research on the effects of monomeric bile acids on the body composition of aquatic animals remains limited. In fish, dietary supplementation with CDCA significantly reduced the hepatic crude lipid content in large yellow croaker [16], as well as the whole-body and muscle crude lipid contents in largemouth bass [23]. However, inconsistent results have been reported for the effect of CDCA on whole-body crude lipid content in Pacific white shrimp [24,25], which may be attributed to differences in supplemental concentration. HDCA supplementation reduced lipid content in the whole body, liver, and muscle of Przevalski’s schizothoracin [20], and a similar decreasing trend in whole-body lipid was observed in the HDCA group in the present study, suggesting a potential lipid-lowering effect. In contrast, UDCA has been reported to increase whole-body and muscle lipid in large yellow croaker [44], and a similar increasing trend was observed in shrimp in this study, indicating a possible lipid-accumulating tendency. Other monomeric bile acids (CA, DCA, and HCA) did not significantly affect body composition in the present experiment.

To date, no studies have reported the effects of monomeric bile acids on the fatty acid composition of aquatic animals. In studies involving mixed bile acids, dietary supplementation with mixed bile acids did not significantly affect the fatty acid composition of the liver and muscle of gilthead seabream (*Sparus aurata*) [37] and black seabream (*Acanthopagrus schlegelii*) [45]. However, in giant freshwater prawn (*Macrobrachium rosenbergii*) [12], although the fatty acid composition of the hepatopancreas was not significantly affected, significant changes were observed in muscle fatty acid composition. Additionally, mixed bile acids induced some alterations in the fatty acid composition of the liver and muscle in tiger puffer (*Takifugu rubripes*) [46]. In the present experiment, none of the monomeric bile acids significantly affected the muscle fatty acid composition of Pacific white shrimp. Based on these findings, the limited effect of monomeric bile acids on muscle fatty acid composition in the present study may be related to the single supplementation level used (0.04%); however, the underlying mechanisms and definitive conclusions require further in-depth investigation.

The reduction in hemolymph TC and TG contents in the CDCA, DCA, and HCA groups may be associated with enhanced lipid transport and clearance. Bile acids function not only as lipid emulsifiers but also as metabolic signaling molecules involved in cholesterol homeostasis. Changes in gene expression related to bile acid transport, sterol metabolism, and lipid catabolism further support enhanced lipid mobilization. Similar lipid-lowering effects of CDCA and DCA have been reported in fish species [22,23], while inconsistent responses have been observed in shrimp and other fish species [23,24,25,43,44]. These results indicate that bile acid effects are compound- and species-dependent and may be influenced by dietary background and supplementation level. Cholesterol plays essential roles in crustaceans, including membrane structure, steroid metabolism, and molting. Therefore, changes in hemolymph lipoproteins may reflect cholesterol redistribution rather than simple lipid accumulation. LDL and HDL are key components of cholesterol transport and reverse cholesterol transport [47,48,49,50,51]. In the present study, lower LDL-C levels in most bile acid groups may indicate reduced cholesterol delivery or enhanced utilization, while higher HDL-C in the UDCA group suggests a distinct regulatory pattern in cholesterol transport. These findings indicate that different monomeric bile acids may regulate cholesterol homeostasis through distinct effects on lipoprotein-mediated transport. In studies on other fish species, UDCA had no significant effect on serum HDL-C and LDL-C contents in Gibel carp [43], but significantly increased the HDL-C content in large yellow croaker [44]. Similarly, CDCA showed no significant effect on the serum HDL-C and LDL-C contents in largemouth bass [23]; however, in Pacific white shrimp [25], dietary supplementation with 600 mg/kg and 900 mg/kg CDCA significantly increased the hemolymph HDL-C and LDL-C contents. In grass carp serum [22], DCA reduced HDL-C and LDL-C contents, whereas CDCA and CA increased their levels (CDCA > CA > DCA). In summary, different monomeric bile acids exerted regulatory effects on the TC, TG, HDL-C, and LDL-C contents. However, these effects exhibited significant species specificity and were influenced by both the type of monomeric bile acids and the supplemental concentration.

CDCA, DCA, and HCA significantly reduced the hemolymph TG levels, while CDCA and HCA reduced the TC levels. In addition, LL levels were higher in all treatment groups compared with the control. Although no significant differences were detected in HL and LL, previous studies in large yellow croaker and Prenant’s schizothoracin have reported increased lipase activities after CDCA or HDCA supplementation [16,20]. These results suggest that monomeric bile acids may influence lipid transport and metabolism in Pacific white shrimp. HL and LPL play critical roles in lipid metabolism. LPL hydrolyzes triglycerides in circulating triglyceride-rich lipoproteins, such as chylomicrons and very-low-density lipoproteins, thereby releasing fatty acids for tissue utilization. HL participates in lipoprotein remodeling by hydrolyzing triglycerides and phospholipids in several lipoproteins, and is involved in HDL and LDL metabolism [52,53]. Therefore, increased HL and LPL activities may contribute to lipid clearance and the reduction in hemolymph TG content. Elevated HL and LL levels enhance their catalytic activity, promoting lipid clearance and consequently reducing the hemolymph TG content. Overall, CDCA, DCA, and HCA showed relatively stronger effects on hemolymph lipid reduction, while CA and HDCA exhibited moderate regulatory effects. UDCA showed a different pattern, characterized by higher HDL-C levels and a numerical increase in whole-body lipid content, suggesting a distinct role in cholesterol transport and lipid redistribution.

The reduction in hepatopancreatic TG and TC in the CA, CDCA, and HCA groups may be associated with enhanced lipid mobilization and metabolic turnover. In crustaceans, the hepatopancreas is the central organ for lipid digestion, storage, and utilization. Thus, changes in lipid content likely reflect direct metabolic regulation by bile acids. In the present study, reduced hepatopancreatic lipid content was accompanied by changes in genes related to sterol transport and lipid catabolism, suggesting enhanced lipid turnover rather than lipid deposition. The lower MDA content observed in all treatment groups indicates a reduction in lipid peroxidation. As MDA is a major end product of lipid peroxidation, its decrease suggests that dietary monomeric bile acids alleviated lipid peroxidation-related oxidative damage in the hepatopancreas. This effect may be associated with the improved lipid metabolic status observed in the present study, including reduced lipid accumulation and enhanced lipid turnover. Similar regulatory effects of monomeric bile acids on lipid metabolism and oxidative status have been reported in fish and shrimp, including largemouth bass, yellow catfish, grass carp, large yellow croaker, and Pacific white shrimp [16,17,22,23,25,42,44,54], highlighting species-specific responses and the influence of bile acid type and level. The enterohepatic circulation constitutes the primary mode of bile acid cycling in vertebrates: bile acids are synthesized in the liver, transported as major constituents of bile to the intestine, reabsorbed by the intestine, and returned to the liver [55]. Kumar et al. [56] reported the existence of a similar enterohepatic circulation in shrimp. Based on this, the present study examined the expression levels of genes related to bile acid uptake (*asbt*, *ostα*) and transport (*mrp2*, *ntcp*). Results showed that the expression levels of *asbt*, *ostα*, *mrp2*, and *ntcp* in the hepatopancreas were higher in all treatment groups compared to the control group. Li et al. [28] observed similar results in studies supplementing mixed bile acids in low-fishmeal diets. The increased expression of genes related to bile acid uptake and transport indicates that dietary monomeric bile acids affected bile acid transport processes in Pacific white shrimp. These changes may contribute to lipid metabolic regulation by facilitating bile acid-mediated lipid digestion, transport, and turnover. Under the single supplementation level used in this study (0.04%), CDCA, DCA, and CA showed relatively stronger regulatory effects on lipid-related biochemical parameters and metabolic gene expression than the other tested bile acids. However, further dose–response studies are required before establishing a definitive efficacy ranking among different monomeric bile acids.

Sterol biosynthesis and its regulation are crucial for lipid metabolism [57]. The genes *7dhcr*, *24dhcr*, *Δ24sr*, *sr*, and *scp* are involved in the synthesis of intracellular sterols or steroid hormones and play essential roles in maintaining sterol homeostasis [58,59,60,61,62]. In this experiment, the expression levels of sterol synthesis-related genes (*24dchr*, *7dhcr*, *Δ24sr*, *sr*, *scp*) were most significantly elevated in the CA and CDCA groups, followed by the DCA group. In contrast, the UDCA, HDCA, and HCA groups showed no significant differences compared to the control group. Thus, it was inferred that CA and CDCA significantly promote sterol production, with DCA also exhibiting a certain promoting effect. Similar results have been reported in studies on the effects of CDCA in Pacific white shrimp [25].

Ldlr mediates the clearance of VLDL and LDL particles from circulation by binding to apolipoproteins Apob100 and Apoe1, thereby reducing cholesterol levels [63,64]. Npc1, an intracellular cholesterol transporter, functions coordinately with NPC2 in the export and excretion of cholesterol from the nucleus or lysosomes [65,66]. In the present study, the expression levels of *ldlr* and *npc1* were highest in the CDCA and CA groups, followed by the DCA and UDCA groups. This pattern aligned with the lower cholesterol content observed in the hepatopancreas of the CA and CDCA groups, indicating that different monomeric bile acids possess varying capacities for cholesterol transport and excretion, with CDCA and CA exhibiting more pronounced effects. Abca1 plays a pivotal role in the efflux of intracellular cholesterol to apolipoproteins and subsequent HDL formation [67]. In this experiment, *abca1* expression in all treatment groups was higher than in the control group, consistent with the observation that hemolymph HDL-C contents were elevated across all treatment groups relative to the control group.

Ampk functions as a critical cellular energy sensor; its activation promotes ATP generation by accelerating fatty acid oxidation and glycolysis while simultaneously inhibiting the synthesis of fatty acids, proteins, and glycogen [68]. Acox is a key enzyme in the fatty acid β-oxidation pathway [69]. Fatp is involved in the uptake and metabolism of long-chain and very-long-chain fatty acids by cells [70]. Fabp preferentially binds long-chain fatty acids and facilitates their transport via FATP, subsequently activating peroxisome proliferator-activated receptors (PPARs) [71]. Pmfe catalyzes the β-oxidation of long-chain fatty acids within peroxisomes [72]. In this experiment, the expression levels of *ampk*, *acox*, *fatp*, *fabp*, and *pmfe* were generally higher in all treatment groups compared to the control group, with relatively higher expression observed in the CA, CDCA, and UDCA groups.

These results indicate that dietary supplementation with monomeric bile acids promotes lipid catabolism, digestion, and absorption in Pacific white shrimp, with CA, CDCA, and UDCA showing more pronounced effects, consistent with previous findings [24,25]. Tgl catalyzes the hydrolysis of intracellular and extracellular triacylglycerols [73]. Elevated *tgl* expression levels are typically associated with more efficient triglyceride hydrolysis, suggesting that the CDCA group in this study may possess higher triglyceride hydrolytic activity. Mttp is responsible for mediating the transport of triglycerides, cholesteryl esters, and phospholipids between phospholipid surfaces and may participate in regulating cholesteryl ester biosynthesis in lipoprotein-synthesizing cells [74,75]. In the present experiment, *mttp* expression was highest in the DCA group, which corresponds with the relatively higher TC content observed in the hepatopancreas of this group. Fad catalyzes the biosynthesis of polyunsaturated fatty acids [76], while Baat regulates intracellular fatty acid metabolism [77]. The CDCA group exhibited the highest expression levels of *fad* and *baat*, with expression levels in nearly all treatment groups exceeding those in the control group, contributing to the maintenance of fatty acid metabolic homeostasis.

These results suggest that dietary monomeric bile acids mainly act as metabolic regulators rather than direct growth promoters under the present dietary condition. The reductions in hemolymph TG, TC, and LDL-C indicate improved circulating lipid transport and cholesterol homeostasis. The lower hepatopancreatic MDA content suggests reduced lipid peroxidation pressure and improved hepatopancreatic status. In addition, the upregulation of genes related to bile acid transport, sterol metabolism, and lipid catabolism indicates enhanced lipid turnover. Therefore, monomeric bile acids may have potential as functional feed additives to regulate lipid metabolism and support hepatopancreatic health in Pacific white shrimp.

## 5. Conclusions

At the tested concentration of 0.04%, dietary monomeric bile acids did not significantly improve growth performance under the present nutritionally adequate dietary condition. However, they altered lipid-related biochemical parameters, reduced the lipid peroxidation marker MDA, and regulated the expression of genes involved in bile acid transport, sterol metabolism, and lipid catabolism, indicating that monomeric bile acids primarily influence metabolic regulation rather than growth performance.

Among the tested compounds, CDCA generally produced the most consistent responses in lipid-related indicators under the present experimental conditions. Because only a single supplementation concentration and one dietary condition were evaluated, these findings should be considered preliminary. Nevertheless, the observed responses suggest that CDCA warrants further investigation, particularly in dietary scenarios where lipid utilization is a major concern, such as high-fat or low-fishmeal diets. Overall, this study demonstrates that monomeric bile acids can modulate lipid metabolism in Pacific white shrimp and provides a basis for future research on the targeted nutritional application of individual bile acid monomers in crustaceans.

## Figures and Tables

**Table 1:** Composition and nutrition contents of the experimental diets (% dry matter).

Ingredient	Control	CA	HDCA	CDCA	DCA	UDCA	HCA
Fish meal ^a^	20	20	20	20	20	20	20
Soybean meal ^a^	33	33	33	33	33	33	33
Peanut meal ^a^	10	10	10	10	10	10	10
Wheat meal ^a^	21.58	21.54	21.54	21.54	21.54	21.54	21.54
Brewer’s yeast ^a^	5	5	5	5	5	5	5
Mineral premix ^b^	0.5	0.5	0.5	0.5	0.5	0.5	0.5
Vitamin premix ^b^	1	1	1	1	1	1	1
Monocalcium phosphate	1	1	1	1	1	1	1
Vitamin C	0.2	0.2	0.2	0.2	0.2	0.2	0.2
Choline chloride	0.2	0.2	0.2	0.2	0.2	0.2	0.2
Betaine	0.3	0.3	0.3	0.3	0.3	0.3	0.3
Ethoxyquin	0.02	0.02	0.02	0.02	0.02	0.02	0.02
Mold inhibitor	0.1	0.1	0.1	0.1	0.1	0.1	0.1
Fish oil ^a^	2	2	2	2	2	2	2
Soya lecithin ^a^	1	1	1	1	1	1	1
Soybean oil ^a^	2	2	2	2	2	2	2
Bile acid ^c^	0	0.04	0.04	0.04	0.04	0.04	0.04
Y_2_O_3_	0.1	0.1	0.1	0.1	0.1	0.1	0.1
Alginate	2	2	2	2	2	2	2
Proximate composition							
Crude protein	43.29	43.05	43.10	43.66	43.16	43.14	43.16
Crude lipid	6.74	6.79	6.84	6.79	6.46	6.74	6.77
Ash	8.66	8.71	8.74	8.80	8.95	8.95	8.88
Moisture	10.05	9.38	9.21	9.50	10.05	9.67	9.07
Gross energy (MJ/kg)	17.84	17.93	18.01	17.98	17.92	17.94	18.05

^a^ Fish meal, soybean meal, peanut meal, wheat flour, brewer’s yeast, fish oil, soybean oil, and soya lecithin (50% purity) were obtained from Qingdao Surgreen Ocean Biological Feed Co., Ltd. (Qingdao, China). The fish meal contained 72.68% crude protein and 6.63% crude lipid (dry matter basis). In the bile acid-supplemented diets, monomeric bile acids were incorporated by replacing 0.04% wheat meal with the corresponding bile acid. ^b^ The vitamin and mineral premixes designed for marine fish were purchased from Qingdao Master Biotech Co., Ltd. (Qingdao, China). The vitamin premix contained a range of vitamins, including retinyl acetate, vitamin D3, DL-α-tocopherol acetate, menadione nicotinamide bisulfite, thiamine, riboflavin, vitamin B6, cyanocobalamin, D-calcium pantothenate, niacinamide, folic acid, D-biotin, L-ascorbate-2-phosphate, inositol, betaine hydrochloride, yeast hydrolysate, and rice hull powder. The mineral premix consisted of ferrous sulfate, zinc sulfate, manganese sulfate, cupric sulfate, cobaltous chloride, sodium selenite, calcium iodate, and zeolite powder. ^c^ Bile acids were purchased from Shanghai Aladdin Biochemical Technology Co., Ltd. (Shanghai, China); purity > 95%.

**Table 2:** Sequences of the PCR primers used in this work.

Function	Gene	Gene Full Name	Forward Primer (5′-3′)	GenBank Reference	PL (bp)
Bile acid metabolism	*asbt*	Sodium/bile acid cotransporter-like	TGGACCCTCATGTTCAACGG	XM_027356442.1	117
			CAACAAACTCGCCCCCAAAG		
	*ostα*	Organic solute transporter subunit alpha-like	CTGGTGACGACCACCTCCTT	XM_027368947.1	59
			GCCTTGAATCCCACGATGAA		
	*mrp3*	Multidrug resistance-associated protein 2-like	GATAGTGACAGGGTGTTAGTCTTGCA	XM_027355584.1	60
			GGCTGGCGTGTCGAATTC		
	*ntcp*	Sodium taurocholate co-transporting polypeptide	CTGTTGCTGTGGCCACCAT	PVHP267466.2	60
			TCCTCTTGGCGATGTATGCA		
Sterol metabolism	*abca1*	ATP-binding cassette sub-family A 1	ACCTGAAGGCAGGACGAAAG	XM_027375794.1	73
			GGCATACTCGCGCTATCTGT		
	*ldlr*	Low-density lipoprotein receptor	CGTCACATGCCCAGCCATAA	XM_027363319.1	86
			GATCCGTCATGGCACTCGAA		
	*amacr*	4F2 cell-surface antigen heavy chain-like	GACAGCTCTGGCGATGTGAATCC	XM_027375138.1	129
			CGAATCGTATGCGGCAGACTCC		
	*hmgcr*	3-Hydroxy-3-methylglutaryl-coenzyme A reductase-like	AGGTGCCCACAAAGACACTC	XM_027354586.1	85
			TGATAGTTCCCCAGCCAGGA		
	*scp*	Sterol carrier protein 2	TCAGAGGAAATGAACGGGGG	XM_027375905.1	148
			TGGAAGCAGTACACACCCTT		
	*24dchr*	24-Dehydrocholesterol reductase	GATCAGCTTTCGGGAGGGAG	XM_027368854.1	112
			CATTGGCTCACATCGCACAG		
	*7dhcr*	7-Dehydrocholesterol reductase-like	AGACCTGTTACGGCTGTTGAG	XM_027377095.1	147
			GACTGGTCGGGACTCCAAAA		
	*Δ24sr*	Delta (24)-sterol reductase-like	TGCTGATTGTGCTACCGCTT	XM_027382756.1	118
			TGAATAGCCCTGACACGCTC		
	*sr*	Steroid reductase	TGCTTGGACCATTCAAGGGG	XM_027383297.1	106
			ACCCGCATAGTCTCTTGTGC		
	*npc1*	NPC intracellular cholesterol transporter 1	CGAAGGGGAAAAGCCAGAGT	XM_027363410.1	87
			TTGAGGAGGAAGGGAGCGTA		
	*npc2*	NPC intracellular cholesterol transporter 2	CGCAGTATCGGCAGTCAAGA	XM_027358057.1	149
			GTGGTGTGAAAGGCAACGAC		
Lipid metabolism	*mttp*	Microsomal triglyceride transfer protein large subunit	GCTGCTAAGGAAAGTGCGTG	XM_027380336.1	215
			AAGGATGCGTCGCTAAGGAG		
	*ampk*	AMP-activated protein kinase subunit beta	TCAGAGGAGGAGCAGGAAC	KP272117.1	88
			CCCGAGGTCTAATAGGCAC		
	*srebp*	Sterol-regulatory element binding protein	AGGCGAGAAACGCAACT	MG770374.1	321
			GAGGGTGATGGAGGCAG		
	*fabp*	Fatty acid binding protein 1-B.1-like	CGCTAAGCCCGTGCTGGAAGT	XM_027377789.1	103
			CTCCTCGCCGAGCTTGATGGT		
	*fatp*	Protein unc-13 homolog 4B-like	TCGTGTCGGTGCTTTTCCC	XM_027375131.1	228
			ATGCGGCGTCTCCTTTCCT		
	*acox*	Peroxisomal acyl-coenzyme a oxidase 1-like	CCCGGGTGTCCACTGACA	XM_027370700.1	100
			GTAGCGTCCGAGGCACTGA		
	*tgl*	Triacylglycerol lipase	ACAAGGTGGATAAGGAAGAG	XM_027365317.1	107
			TAATCAGTAGTTGGCGAAGA		
	*fad*	Delta6 fatty acyl desaturase	TCCTGGTATTGGGAGTGA	KT305965.1	123
			ACCTCTACCCAGCGTTCT		
	*acbp*	Acyl-CoA-binding protein	ATCCCACTTTGCCTTTCC	MK840980.1	119
			AGCAGCCAACTGATGACG		
	*pmfe*	Peroxisomal multifunctional enzyme type 2-like	AAGTTTGGCCGCATCATCAT	XM_027370242.1	100
			TCAAGCCAAGCAGACCAAGTT		
	*baat*	Acyl-coenzyme A thioesterase 1-like	CACTCCGAGTTCTTGCCTTCA	XM_027379581.1	100
			GGTATCCTTCATGGACGAGTACAGA		
Reference gene	*ef-1α*	*Elongation Factor 1-alpha*	GTATTGGAACAGTGCCCGTG	GU136229.1	143
			ACCAGGGACAGCCTCAGTAAG		
	*β-actin*	*Beta-actin*	CGAGGTATCCTCACCCTGAA	AF300705.2	176
			GTCATCTTCTCGCGGTTAGC		

**Table 3:** Growth performance of experimental shrimp (mean ± standard error).

Parameter	Control	CA	HDCA	CDCA	DCA	UDCA	HCA
IBW (g)	2.51 ± 0.06	2.46 ± 0.02	2.54 ± 0.06	2.52 ± 0.01	2.62 ± 0.07	2.53 ± 0.07	2.56 ± 0.01
FBW (g)	11.45 ± 0.36	10.87 ± 0.19	11.19 ± 0.17	10.73 ± 0.34	11.55 ± 0.20	11.33 ± 0.22	11.32 ± 0.13
WG (g)	8.95 ± 0.30	8.39 ± 0.21	8.65 ± 0.12	8.22 ± 0.33	8.93 ± 0.23	8.80 ± 0.26	8.76 ± 0.13
WGR (%)	356.48 ± 5.18	341.04 ± 11.79	341.40 ± 4.60	326.52 ± 11.92	341.27 ± 14.34	348.28 ± 18.45	341.37 ± 4.45
SGR (%/day)	2.71 ± 0.02	2.65 ± 0.05	2.65 ± 0.02	2.59 ± 0.05	2.65 ± 0.06	2.68 ± 0.07	2.65 ± 0.02
FCR	1.12 ± 0.04	1.09 ± 0.04	1.10 ± 0.04	1.23 ± 0.06	1.15 ± 0.02	1.11 ± 0.02	1.14 ± 0.06
SR (%)	0.92 ± 0.01	0.97 ± 0.02	0.97 ± 0.02	0.93 ± 0.02	0.91 ± 0.02	0.96 ± 0.01	0.95 ± 0.02

IBW, initial body weight; FBW, final body weight; WG, weight gain; WGR, weight gain ratio; SGR, specific growth rate; FCR, feed conversion ratio; SR, survival.

**Table 4:** Body proximate composition of whole shrimp and muscle fed with diets containing different bile acids (%wet weight, mean ± standard error).

Parameter	Control	CA	HDCA	CDCA	DCA	UDCA	HCA
Whole shrimp							
Moisture	74.42 ± 0.09	74.51 ± 0.69	74.42 ± 0.53	75.16 ± 0.58	74.61 ± 0.14	73.68 ± 0.36	73.68 ± 0.33
Crude protein	19.22 ± 0.18	19.26 ± 0.45	19.09 ± 0.17	18.70 ± 0.33	19.19 ± 0.22	19.44 ± 0.34	19.65 ± 0.42
Crude lipid	1.91 ± 0.27	1.87 ± 0.18	1.74 ± 0.30	2.11 ± 0.05	2.01 ± 0.20	2.37 ± 0.04	1.96 ± 0.29
Ash	2.90 ± 0.05	2.89 ± 0.05	2.92 ± 0.03	2.75 ± 0.11	2.78 ± 0.05	2.96 ± 0.06	3.6 ± 0.12
Muscle							
Moisture	73.78 ± 0.23	73.92 ± 0.23	73.49 ± 0.34	73.35 ± 0.28	73.95 ± 0.04	73.48 ± 0.52	74.44 ± 0.07
Crude protein	23.35 ± 0.09	23.07 ± 0.19	23.55 ± 0.23	23.41 ± 0.26	23.08 ± 0.06	23.54 ± 0.31	22.86 ± 0.12
Crude lipid	1.10 ± 0.03	1.11 ± 0.03	1.13 ± 0.04	1.21 ± 0.05	1.12 ± 0.03	1.12 ± 0.04	1.04 ± 0.03

**Table 5:** Muscle fatty acid composition of experimental shrimp with diets containing different bile acids (%TFA, mean ± standard error).

Fatty Acid	Control	CA	HDCA	CDCA	DCA	UDCA	HCA
C14:0	0.23 ± 0.06	0.26 ± 0.04	0.23 ± 0.04	0.25 ± 0.04	0.23 ± 0.03	0.21 ± 0.01	0.23 ± 0.04
C16:0	19.23 ± 0.12	19.52 ± 0.11	19.20 ± 0.22	19.48 ± 0.09	19.45 ± 0.16	18.73 ± 0.28	19.22 ± 0.09
C18:0	9.34 ± 0.22	9.68 ± 0.17	9.79 ± 0.22	9.54 ± 0.24	9.77 ± 0.06	9.68 ± 0.09	9.04 ± 0.43
C20:0	0.23 ± 0.01	0.23 ± 0.01	0.22 ± 0.01	0.23 ± 0.00	0.22 ± 0.01	0.22 ± 0.00	0.20 ± 0.01
∑SFA	29.02 ± 0.06	29.68 ± 0.14	29.44 ± 0.11	29.50 ± 0.23	29.67 ± 0.20	28.85 ± 0.18	28.69 ± 0.33
C14:1n-5	0.16 ± 0.00	0.15 ± 0.01	0.16 ± 0.00	0.16 ± 0.01	0.16 ± 0.00	0.15 ± 0.01	0.19 ± 0.03
C16:1n-7	1.04 ± 0.13	1.07 ± 0.07	0.95 ± 0.05	0.99 ± 0.07	0.92 ± 0.01	0.93 ± 0.04	0.98 ± 0.10
C18:1n-9	13.37 ± 0.37	13.41 ± 0.14	12.92 ± 0.27	13.02 ± 0.33	12.62 ± 0.13	12.90 ± 0.35	12.51 ± 0.17
C22:1n-9	0.16 ± 0.00	0.17 ± 0.01	0.16 ± 0.01	0.16 ± 0.01	0.16 ± 0.01	0.17 ± 0.01	0.15 ± 0.00
∑MUFA	14.74 ± 0.51	14.81 ± 0.21	14.18 ± 0.32	14.32 ± 0.40	13.87 ± 0.15	14.14 ± 0.40	13.82 ± 0.26
C18:2n-6	15.41 ± 0.28	15.48 ± 0.09	14.96 ± 0.20	15.26 ± 0.36	14.75 ± 0.22	15.20 ± 0.12	15.12 ± 0.83
C20:2n-6	1.49 ± 0.05	1.57 ± 0.03	1.61 ± 0.04	1.51 ± 0.01	1.56 ± 0.03	1.61 ± 0.10	1.51 ± 0.04
∑n-6 PUFA	18.39 ± 0.24	18.52 ± 0.10	18.15 ± 0.17	18.31 ± 0.30	17.95 ± 0.20	18.40 ± 0.18	18.37 ± 0.75
C18:3n-3	0.83 ± 0.05	0.85 ± 0.02	0.78 ± 0.01	0.82 ± 0.03	0.76 ± 0.02	0.81 ± 0.04	0.74 ± 0.06
C20:3n-3	1.50 ± 0.07	1.47 ± 0.02	1.58 ± 0.01	1.54 ± 0.08	1.63 ± 0.05	1.60 ± 0.05	1.73 ± 0.09
C20:5n-3	10.99 ± 0.40	10.48 ± 0.21	11.46 ± 0.06	10.99 ± 0.56	11.73 ± 0.18	11.39 ± 0.32	11.42 ± 0.27
C22:5n-3	0.84 ± 0.06	0.87 ± 0.09	0.79 ± 0.02	0.74 ± 0.02	0.75 ± 0.02	0.80 ± 0.04	0.75 ± 0.04
C22:6n-3	12.43 ± 0.15	12.22 ± 0.28	12.54 ± 0.26	12.33 ± 0.25	12.71 ± 0.08	12.86 ± 0.22	12.85 ± 0.30
∑n-3 PUFA	26.59 ± 0.45	26.19 ± 0.27	27.15 ± 0.27	26.43 ± 0.83	27.57 ± 0.26	27.46 ± 0.43	27.49 ± 0.84
∑n-3/∑n-6	1.37 ± 0.04	1.33 ± 0.02	1.41 ± 0.01	1.36 ± 0.06	1.45 ± 0.03	1.41 ± 0.03	1.41 ± 0.10

SFA: saturated fatty acid; MUFA: monounsaturated fatty acid; PUFA: polyunsaturated fatty acid.

## Data Availability

The raw data supporting the conclusions of this article will be made available by the authors on request.
